# Evaluation of diagnostic performance of non-invasive HIV self-testing kit using oral fluid in Addis Ababa, Ethiopia: A facility-based cross-sectional study

**DOI:** 10.1371/journal.pone.0210866

**Published:** 2019-01-25

**Authors:** Wudinesh Belete, Tekalign Deressa, Altaye Feleke, Takele Menna, Tezera Moshago, Saro Abdella, Abebe Hebtesilassie, Yimam Getaneh, Minilik Demissie, Yonas Zula, Israel Lemma, Girmachew Mamo, Endale Workalemahu, Tsigereda Kifle, Ebba Abate

**Affiliations:** 1 Ethiopian Public health institute, Addis Ababa, Ethiopia; 2 Population service International/E, Addis Ababa, Ethiopia; Instituto Rene Rachou, BRAZIL

## Abstract

**Background:**

Human immunodeficiency virus (HIV) testing is critical for early linkage to treatment and care services. However, there is a substantial gap in HIV testing, particularly in resource limited settings due to low accessibility of HIV testing sites, inconvenient testing hours, and concerns about loss of confidentiality. Thus, adopting new strategies such as HIV self-testing (HIVST) could overcome these barriers and increases HIV testing uptake.

**Objective:**

The aim of this study was to evaluate the diagnostic performance of non-invasive HIVST kit using oral fluid for HIV diagnosis. This study also aimed to assess the ability of clients in interpretation of OraQuick HIVST results.

**Method:**

Between December 2017 and February 2018, a total of 400 study participants were enrolled into the study to assess a diagnostic accuracy of Oral fluid-based HIVST kit (OraQuick) in 15 public health facilities in Addis Ababa, Ethiopia. Participants were provided with instructions and visual aids on how to perform HIVST and interpret results. They also underwent a blood-based rapid HIV antibody test as per the current national algorithm. The results of HIVST were interpreted independently by the participants and respective health care workers (HCWs). The sensitivity, specificity, positive predictive value (PPV), Negative predictive value (NPV) and inter-rater agreement of the test were computed.

**Results:**

Out of 200 participants who tested positive on the national algorithm testing, oral fluid-based self-testing was positive in 199 (99.5%), false negative in 1 (0.5%). Of 200 participants who tested negative on the national algorithm testing, self-testing was negative in 200 (100%). There were no false positive and invalid tests. The sensitivity and specificity of the OraQuick HIVST were 99.5% (95%CI: 97.26–99.99) and 100% (95%CI: 98.18–100.0), respectively. The overall agreement between the two tests was high (κ value = 0.995). The PPV and NPV of OraQuick test were 100% and 99.5% (95%CI: 96.59–99.93) respectively.

**Conclusion:**

This study showed a high diagnostic performance of OraQuick HIV self-test and suggests that OraQuick HIVST kit has a potential to be used for HIV testing in Ethiopia along with the national algorithm.

## Background

Despite the significant progress towards curbing the epidemic, HIV continues to be a public health threat. Since its recognition, HIV has claimed over 34 million lives worldwide and about 36.7 million people were living with HIV by the end of 2017. The burden of HIV is particularly high in Sub- Saharan Africa where about 76% of the world HIV/AIDS infections and deaths occur [1].

The current global strategy for high impact HIV prevention emphasizes the importance of enhanced HIV-testing services (HTS) targeting high-risk persons as a key measure for program evaluation [2, 3]. HIV testing is critical for early linkage to treatment and care services, partner prevention services, and focused behavioral interventions [2, 4]. However, coverage of HIV testing remains low, and a significant number of people with HIV are still unaware of their status [5–7]. In resource limited settings, factors like low accessibility of HIV testing sites, inconvenience of testing hours, concerns about loss of confidentiality, and lack of confidence in the competence of health personnel have been associated with the low HIV testing uptake [8, 9]. One approach to overcome such barriers to HIV testing is an introduction of HIVST strategy [10, 11]. HIVST allows individuals to learn their HIV status earlier before they become sick, bring services closer to where they live, and create demand for HIV testing [12]. This strategy is particularly important for those people facing barriers to accessing existing services [13–15].

Currently, Ethiopia is planning to introduce HIVST as a complementary approach to the existing facility based HTS, with the aim of reaching previously untested, hard-to-reach and test-averse populations. Prior research revealed that HIVST was widely accepted among users in various settings and increased the frequency of HIV testing, particularly in key population [14–17]. For example, in a community-based study in Malawi, 92% of participants opted for supervised self-testing over standard HTC [17]. Similarly, another study conducted in Kenya found that HIVST had increased HIV testing among male partners when compared with standard testing [16].

Previous studies conducted to validate the performance of the OraQuick Rapid HIV-1/2 test found a comparable accuracy of HIVST with the conventional blood-based HIV rapid tests [18–20]. However, to date, there is no empirical evidence on the performance of HIVST in Ethiopia to support the implementation of this strategy. The World Health Organization (WHO) recommends evaluating the diagnostic performance of each kit to determine its accuracy and suitability in the context of a given country setting before use [21]. To this end, we assessed the sensitivity, specificity, PPV and NPV of oral fluid-based OraQuick kit (OraSure Technologies Inc, PA, USA) for HIV diagnosis at selected health facilities in Addis Ababa, Ethiopia. Further, we assessed the ability of clients in interpretation of OraQuick HIVST results.

## Material and methods

### Assay kit

OraQuick HIV-1/2 kit is one of the innovative self-test kits recommended by the WHO. It is a visually read, qualitative immunochromatographic test for the detection of antibodies to HIV-1/2. It is an easy to use kit that has a potential to be used for surveillance purposes, in antenatal care clinics, and in hard-to-reach areas [22]. Moreover, this technology can also reduce the risk of accidental exposure to HIV through contaminated blood and reduce the need for disposal of biohazardous waste.

### Study design and setting

This facility based cross-sectional study was conducted at 15 public health facilities in Addis Ababa. Data were collected between December 2017 and February 2018. Health facilities for the OraQuick HIVST kit diagnostic performance evaluation were chosen purposively in advance based on their high HIV positive yields and logistic considerations.

### Study population and participants recruitments

This study targeted community members who visited the health facilities for voluntary counseling and testing (VCT) or provider-initiated HIV counseling and testing (PICT), and prevention of mother-to-child transmission of HIV (PMTCT) clinics during the study period. Study participants who fulfilled the following eligibility criteria were selected: age over 18 years, and clients who know their HIV positive serostatus if they did not initiate ART. However, clients were excluded from participation if they had bleeding gum, and/or critically ill.

A sample size for this study was determined based on a guideline for evaluation of HIV testing technologies in Africa [21]. According to this guideline, a minimum of 200 HIV-positive and 200 HIV-negative specimens are required to provide 95% confidence intervals (CI) of less than ± 2% for the estimated sensitivity (99%) and specificity (98%). Therefore, this study was conducted among 400 consecutive study participants from fifteen high HIV positive yield public health facilities in Addis Ababa. Ethical clearance for this study was obtained from the Scientific and Ethical Research Office (SERO) of the Ethiopian Public Health Institute (IRB Ref. No: EPHI-IRB-056-2017). All study participants gave a written informed consent prior to data collection. The interviews with study participants were conducted with strict privacy and confidentiality.

### Data collection tool

Structured questionnaire was used to collect information on socio-demographic and clinical risk factors. Variables were chosen for inclusion based on their clinical relevance with the outcome variable being HIV serostatus and independent variables included age, sex, marital status, residence, occupation, history of alcohol consumption, risk behaviors to HIV and other sexually transmitted infections.

### Study procedure

**Fig 1:**
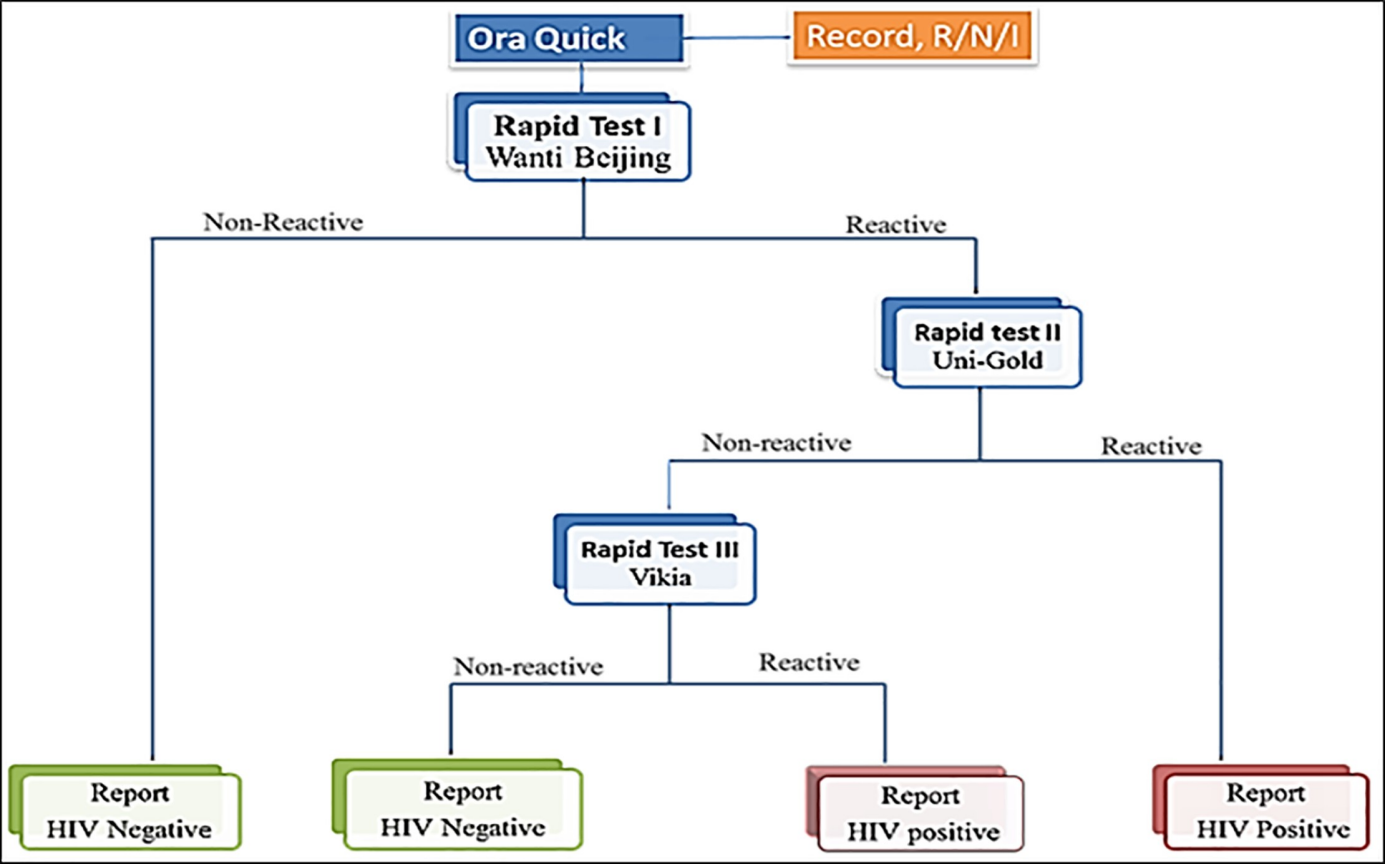
HIV testing algorithm used in this study.

As per the national guideline for HIV testing, pre-test counseling was provided to each study participant who consented to be enrolled to this study. Following the counseling, participants’ sociodemographic and other relevant information were collected by a trained health care provider through one-to-one interview using structured questionnaire. Then, the counsellors explained the OraQuick HIVST procedure to each participant and demonstrated how to conduct the test and interpret the results as per the manufacturer’s instructions. Further, the participants were provided with visual model illustrating test performance and results interpretations (as positive, negative and an invalid). Following the demonstration, each participant performed HIVST using oral swab under supervision of health professionals. Further, participants received a blood-based rapid diagnostic test (RDT) per the national algorithm for HIV testing (Wantai, Uni-Gold and Vikia) “Fig 1”. The HIVST results were interpreted by participants first in a private setting and by health professionals independently at the recommended time-points (20–40 minutes). OraQuick test results were recorded as reactive (R) or non-reactive (N) or Invalid (I) as interpreted either by the client or respective health professional. The result by RDTs per the national algorithm was considered as a final and disclosed to the participants. As part of the routine procedure, all clients who were found to be HIV positive per the national algorithm were linked to HIV care and treatment services.

### Data quality control

Prior to data collection, training on the study procedure was provided to data collectors (two from each health facility) at Ethiopian public health institute. Further, close follow-up and supportive supervision were given throughout the study period. Dried blood spot (DBS) samples were also collected for external quality assessment (EQA) of the OraQuick HIVST kit performance. During the supervision, the supervision team reviewed documentations, adherence to the protocol, and discarded incomplete questionnaires & data with incorrect DBS samples from the study.

### Data analysis

Data was entered, cleaned and analyzed using Epi Info.7 (CDC, Atlanta, GA 30329–4027 USA). Descriptive statistics including percentages, median, and ranges were applied as appropriate. Estimates of sensitivity, specificity, PPV, and NPV with 95% CI for each index test and read condition was computed by comparing results of the OraQuick kits against the reference diagnostic algorithm. Further, analysis of the agreement between OraQuick kit and the reference was conducted by computing a Kappa statistic [23]. P-values <0.05 were considered statistically significant.

## Results

From December 2017 to February 2018, a total of 400 participants were enrolled to the study to evaluate the diagnostic performance of OraQuick HIVST kit. Demographic characteristics of the participants are presented in Table 1. About 60.8% (243/400) of the study participants were female, and the median age was 29 years (IQR: 17.7–40.3). Overall, 54.8% of participants had attended secondary school and above, and 12.3% had no formal education.

**Table 1:** Demographic characteristics of the study participants.

	Characteristics	Frequency	Percent (%)
**Sex**	Male	142	35.5
	Female	243	60.8
	Missing	15	3.8
**Age Group (years)**	≤25	101	25.3
	26–35	174	43.5
	>35	111	27.8
	Missing	14	3.5
**Education**	No formal education	49	12.3
	Elementary	111	27.8
	Secondary	140	35.0
	College and above	79	19.8
	Missing	21	5.3
**Marital Status**	Never Married	107	26.8
	Married	219	54.8
	Divorced/Widowed	56	14.0
	Missing	18	4.5
**Occupation**	Government employee	58	14.5
	Housewife	91	22.8
	Merchant	40	10.0
	Daily laborer	78	19.5
	Students	31	7.8
	Others*	102	25.5

*Others: private/self-employed, drivers, and engineers

### Sensitivity and specificity of OraQuick HIVST kit

All of the study participants underwent both oral fluid-based OraQuick self-testing and conventional blood-based HIV antibody rapid tests as per the national algorithm “Fig 1”. Out of 200 participants who tested positive on the national algorithm testing, oral fluid-based self-testing was positive in 199 (99.5%), false negative in 1 (0.5%). Of 200 participants who tested negative on the national algorithm testing, self-testing was negative in 200 (100%) and positive in 0 (0.0%). There were no invalid tests, but two tests were rejected due to errors (spilling test buffer) in performing the test and were not include into the analysis. The sensitivity and specificity of the OraQuick test were 99.5% (95%CI: 97.26–99.99) and 100.0% (95%CI: 98.18–100.00), respectively when compared to the national algorithm. The overall agreement between the two tests was high (κ value = 0.995). The PPV and NPV of OraQuick test were 100% and 99.5% (95%CI: 96.59–99.93) respectively (Table 2).

**Table 2:** Sensitivity and specificity of OraQuick HIVST kit.

	Percent (95% CI)
**Sensitivity**	99.5(97.26–99.99)
**Specificity**	100.0(98.18–100.00)
**PPV**	100.00
**NPV**	99.5(96.59–99.93)
**Accuracy**	99.75(98.62–99.99)

CI: confidence interval; PPV: Positive Predictive value; NPV: Negative predictive value

This study also explored the ability of the study participants to interpret the OraQuick HIVST results. All of the study participants correctly interpreted negative results in this study. However, one participant misinterpreted a positive result as a negative (Table 3).

**Table 3:** Patterns of OraQuick self-test interpretation by participants and health care workers (HCWs).

HCWs	Participants	Frequency (%)
**Positive (n = 200)**	Positive	199 (99.5)
	Negative	1.0(0.5)
	Invalid	0.0(0.0)
**Negative (n = 200)**	Positive	0.0(0.0)
	Negative	200(100)
	Invalid	0.0(0.0)
**Total**		400(100)

## Discussion

The finding of this study demonstrates high sensitivity and specificity of oral fluid-based OraQuick HIV-1/2 antibody test. It also showed a high rate of agreement between OraQuick test and the national HIV testing algorithm (κ = 0.995). The error rate identified in this study was negligible (0.25%); only a single disagreement was noted between OraQuick HIVST and the national algorithm testing out of 400 HIV tests.

The high sensitivity and specificity of OraQuick HIVST in our study setting was in agreement with the findings of other studies reported from sub-Saharan Africa, and different parts of the world [18–20, 24]. For example, the study conducted in Zambia using OraQuick kit showed a sensitivity of 98.7% (95%CI, 97.5–99.4) and specificity of 99.8% (95%CI, 99.6–99.9) [24]. A similar study conducted in Singapore reported a sensitivity of 97.4% (95% CI: 95.1–99.7) and specificity of 99.9%, (95% CI: 99.6–100) [25]. Therefore, the findings of this study confirm and further expand the excellent diagnostic performance of OraQuick HIVST kit.

In this study, we found one false negative case by OraQuick test. Evidence from post-test counseling showed that this client had multiple histories of treatment for sexually transmitted infections. Thus, the observed false negative test could be due to some interference of the preexisting condition(s) with the OraQuick test. In fact, this finding was in line with the limitation reported by the manufacturer stating that OraQuick often gives inaccurate results in ART patients, pregnant women, and those with some infections like viral hepatitis [26]. When compared with other studies, the observed error rate (0.25%) in this study was lower than 0.4% reported from Malawi [17] and 3.2% from Singapore [25].

Beyond evaluating the diagnostic accuracy of OraQuick HIVST kit, this study also sought to assess the participants’ ability to interpret test results as compared to healthcare workers. Our results showed that 399/400 participants accurately interpreted the test results. Only one client, who knew his HIV positive status, misinterpreted positive result as a negative. Moreover, during the supervision of OraQuick evaluation, we noticed a high level of users’ supports for the HIVST approach. Nevertheless, issues related to the feasibility and acceptability of OraQuick kit remains the subject of further investigation. Overall, these findings suggest that OraQuick HIVST kit has a potential to be used in parallel with the conventional HTS in Ethiopia owing to its higher diagnostic accuracy; and simplicity in conducting the test and interpreting test results by non-clinical users. This HIV testing approach might avoid the barrier to the conventional blood-based HTS and increase HIV testing uptake.

## Limitations

This validation study was conducted in an urban setting, in Addis Ababa. This may limit the generalizability of the results to the wider rural population, especially in terms of conducting HIVST and result interpretation due to variation in education status between the two settings. Further, this study was conducted under the supervision of HCWs which might help the beneficiaries to get additional information in conducting HIVST and result interpretation. This could introduce a possibility for overestimation of the sensitivity and specificity of the self-testing kit as well as the clients’ accuracy rates in results interpretation. In line with this, a previous study conducted in a private setting found a lower accuracy of OraQuick test and high rates of misinterpretation [27]. In this study, we used the national HIV testing algorithm as a standard. Therefore, the sensitivity and specificity of OraQuick HIV test noted in this study were dependent on the accuracy of the standard used. Furthermore, we also included a significant number (5%) of known HIV-positive cases, and it is likely that prior knowledge of their status might have affected the way they interpreted the OraQuicktest result and could contribute to an overestimation of interpretation accuracy.

## Conclusion and recommendation

This study found a high diagnostic performance of OraQuick HIVST kit in our setting. Moreover, the study participants showed no notable problem in conducting the OraQuick HIVST and interpretation of the results. Therefore, although more data on feasibility, acceptability, and extents of linkage to service following HIVST is needed, our finding shows that OraQuick HIVST kit has a potential to be used as a complementary approach for HIV testing in Ethiopia.

## Supporting information

S1 File Questionnaire for OraQuick performance evaluation. (PDF)
