# Supplementary material for: Evaluation of diagnostic performance of non-invasive HIV self-testing kit using oral fluid in Addis Ababa, Ethiopia: A facility-based cross-sectional study
Source: PLoS One. 2019 Jan 25;14(1):e0210866. doi: 10.1371/journal.pone.0210866 (PMC6347142; doi:10.1371/journal.pone.0210866)
Supplement: S1 File — (PDF) [file pone.0210866.s001.pdf]

Questionnaire to assess performance of OraQuick for diagnosis of HIV among clients attending VCT/PICT and ART clinics in Addis Ababa

Code No ___________________

A. Demographics

1. Age__________
2. Sex_____________
3. Educational status A. Illiterate B. Elementary C. Secondary D. College/University
4. Residence: A. Urban B. Rural
5. Religion A. Orthodox B. Muslim C. Protestant D. Other/specify____________
6. Marital Status: A. Married B. never married C. Divorced/separated D. Widowed
7. Occupation A. Government employee B. Housewife C. Student D. Merchant E. daily laborer F. Farmer G. deriver F. Other/Specify_________
8. Monthly in come________________________________

B. Explanatory variables

**Variables**	**Yes**	**No**
History of blood transfusion		
Tooth/body tattooing		
History of unsafe injection		
History of tooth extraction		
History of surgery		
History of sharing sharp objects (Needles, razor blade etc)		
History of treatment for liver diseases		
Do you have family member infected/treated for HBV/HCV		
History of treatment for STIs (syphilis, gonorrhea, chancroid, etc).		
Alcohol use		
Multiple life time sexual partner (please indicate the number, if yes)_________________		
